# Dietary Green-Algae *Chaetomorpha linum* Extract Supplementation on Growth, Digestive Enzymes, Antioxidant Defenses, Immunity, Immune-Related Gene Expression, and Resistance to *Aeromonas hydrophila* in Adult Freshwater Snail, *Bellamya bengalensis*

**DOI:** 10.3390/ani16020289

**Published:** 2026-01-16

**Authors:** Hairui Yu, Govindharajan Sattanathan, Mansour Torfi Mozanzadeh, Pitchai Ruba Glory, Swaminathan Padmapriya, Thillainathan Natarajan, Ramasamy Rajesh, Sournamanikam Venkatalakshmi

**Affiliations:** 1 Key Laboratory of Coho Salmon Culturing Facility Engineering, Institute of Modern Facility Fisheries, College of Biology and Oceanography, Weifang University, Weifang 261061, China; 2 Department of Zoology, St. Joseph University, Chumoukedima 797115, Nagaland, India; 3 Department of Aquaculture, South Iran Aquaculture Research Centre, Iranian Fisheries Science Research Institute (IFSRI), Agricultural Research Education and Extension Organization (AREEO), Ahwaz 314661861, Iran; mansour.torfi@gmail.com; 4 Department of Zoology, M. R. K. College of Arts and Science, Pazhanchanallur, Kattumannarkoil 608301, Tamilnadu, India; slotraufrubaglory@gmail.com; 5 Department of Zoology, Government College for Women (Autonomous), Kumbakonam 612001, Tamilnadu, India; spsrijan23@gmail.com (S.P.); dr.s.venkatalakshmi@gcwk.ac.in (S.V.); 6 Department of Biotechnology, Faculty of Science and Humanities, SRM Institute of Science and Technology, Kattankulathur, Chennai 603203, Tamilnadu, India; nndearar@gmail.com; 7 Department of Zoology, Sacred Heart Arts and Science College, Perani, Villupuram 615202, Tamilnadu, India; rajdhivo@gmail.com

**Keywords:** gastropods, growth performance, macro algae, mucus immunity, freshwater snail

## Abstract

Macroalgae plays a significant role in the formulation of innovative and environmentally sustainable approaches to address food challenges. Specifically, green macroalgae serve as dietary supplements aimed at improving the health, growth, and feeding efficiency of various species of marine and freshwater fishes, as well as mollusks. The effects of *Chaetomorpha linum* extract (CLE) on growth performance, physiological responses, and disease resistance are studied in *Bellamya bengalensis* against *Aeromonas hydrophila*. In this experiment, adult *B. bengalensis* (4412 ± 165.25 mg) were randomly divided into 15 rectangular glass aquariums (35 snail/aquaria; 45 L capacity) and their basal diet was supplemented with different levels of CLE, including 0 (CLE0), 1 (CLE1), 2 (CLE2), 3 (CLE3), and 4 (CLE4) g/kg for 60 days. The growth performance in the CLE3 dietary group was significantly higher that of the CLE0 group, exhibiting both linear and quadratic trends in relation to dietary CLE levels (*p* < 0.05). The activities of pepsin, amylase, and lipase were found to be highest in CLE3 and lowest in CLE0. Both linear and quadratic responses to dietary CLE levels in digestive enzymes were observed (*p* < 0.05). The activities of superoxide dismutase and catalase in the hepatopancreas were found to be elevated in snails due to the synergistic effect of the supplemented CLE diet. Among different levels of diet given, CLE2-supplemented snails showed an increase in their enzyme activity (*p* < 0.05). Interestingly, all the CLE-treated snails expressed elevated levels of mucus lysozyme and mucus protein when compared to control (*p* < 0.05). Additionally, hepatopancreatic acid phosphatase and alkaline phosphatase activity were elevated in snails consuming CLE3 (*p* < 0.05). The transcription levels of immune-related genes, including *mucin-5ac* and *cytochrome*, were significantly elevated in snails that were fed a diet supplemented with 2–4 g of CLE/kg. Furthermore, the transcription level of the *acid phosphatase-like 7 protein* gene also increased in snails receiving CLE-supplemented diets. After a 14-day period of infection, snails that consumed a diet supplemented with 3–4 g/kg of CLE exhibited a notable increase in survival rates against virulent *A. hydrophila*. Based on the above findings, it is suggested that a diet supplemented with 3 g/kg of CLE may enhance growth, antioxidant and immune defense, and disease resistance in the freshwater snail *B. bengalensis*.

## Simple Summary

Algae represent a naturally abundant source of nutrients and serve as the primary food producers in the aquatic food chain for animal life. The cultivation of algae is characterized by its eco-friendly nature and non-toxicity levels. Algae possess numerous advantageous properties, including immunostimulant, antioxidant, anti-inflammatory, and anti-microbial activities in aquatic animals. There is no evidence to support the effectiveness of artificial or natural feeds as substitutes for *Chaetomorpha* spp. algae in the rearing of freshwater gastropod mollusks. This study investigates the effects of incorporating *Chaetomorpha linum* extract (CLE) into the diet of gastropods, revealing enhancements in growth, immune response, levels of digestive enzymes, and antioxidant activity. Furthermore, various concentrations of CLE extract in the diet may positively influence the growth of freshwater gastropod mollusks, particularly in their resistance to bacterial infections.

## 1. Introduction

Farmed mollusks are playing an increasingly vital role in the global food supply. In 2020, aquaculture accounted for approximately half of the world’s aquatic animal production, with the other half sourced from capture fisheries [1]. In 2022, global aquaculture production of mollusks reached 17.7 million tonnes, valued at around 29.8 billion USD [1]. The majority of molluscan aquaculture consists of non-cephalopod mollusks, particularly marine bivalves such as scallops, clams, oysters, and mussels. In certain countries, marine bivalves are cultivated more than other aquatic species. For instance, marine bivalves represent 86.9% of aquaculture in New Zealand, 75.4% in France, and 74.8% in Spain [1]. The freshwater edible snail (*Bellamya bengalensis*) is a gastropod species from the Mollusca phylum, Gastropoda class, Mesogastropoda order, and Viviparidae family. It is widely distributed across Asia and Africa. Europe, mostly the Netherlands, France, and Austria, has historically valued the traditional delicacies of freshwater mollusks. For tribal groups in Bangladesh and India, in particular, these freshwater mollusks, primarily *B. bengalensis*, provide a sustainable and economical alternative to animal protein. This snail meat has a low fat and cholesterol level and is rich in nutrients and essential amino acids, and is well liked for its tasty and delicate cooking [2]. Snail flesh has been used for its anti-inflammatory, immune-boosting, anti-microbial, antioxidant, and anti-hypertensive properties [3,4]. A cheap source of protein is used to make bioactive peptides with nutraceutical uses. Using enzyme technology, the current study extracted and characterized bioactive peptides from the meat of *B. bengalensis* snails [2,5]. Until recently, a range of snails, including *Archatina archatina*, *A. fulica*, and *Babylonia spirata*, have been efficient at suppressing bacterial infections [6,7,8]. Due to their lack of acquired immunity, mollusks mainly rely on innate immunity and bioactive substances for healing wounds in the microbe-rich marine ecosystem and defense against microbial infections [9]. Cultured mollusks are susceptible to a variety of pathogens, including viruses, bacteria, fungi, and parasites. The Gram-negative motile bacterium *Aeromonas hydrophila* is a prime example of such an opportunistic pathogen. It is one of the most prevalent aquatic diseases, affecting a wide range of aquatic animals [10].

Seaweeds have long served as a vital source of nutrition in various Asian continents, but their potential for medicinal and pharmaceutical uses has only recently come to light on a global scale [11]. Numerous seaweeds contain bioactive compounds, which are extensively documented in the scientific literature, demonstrating substantial efficacy as anti-microbial, anti-inflammatory, anti-cancer, and anti-diabetic agents [12,13,14]. Algal nutrition plays a crucial role in the survival and growth of mollusk larvae. Both micro- and macroalgal diets offer essential nutrients that satisfy the metabolic demands necessary for the growth and reproduction of mollusks [15,16]. Since gastropods and bivalve shellfish are unable to synthesize essential fatty acids during their growth, they must obtain these vital nutrients from external sources [17,18]. It is anticipated that algae will remain the most effective food source for nurseries and farms raising gastropods and bivalve shellfish, even with the forthcoming introduction of synthetic bait and artificial feed options [19,20]. Research indicates that juvenile abalone (*Haliotis squamata*) exhibit faster growth rates when their diets include *Spirulina platensis* compared to a diet containing only 2% algae [21]. Furthermore, *Pila globosa* that received a diet with a combination of *Eichhornia* or *Vallisneria* macrophytes demonstrated enhanced growth and feed efficiency [22]. The genus *Chaetomorpha* (Chlorophyta, Cladophorales) is defined by its unbranched, robust filaments [23] and includes 70 species [24]. Many of these species are known to contain bioactive compounds that allow for a range of applications [25]. Some species have been studied to identify the chemical nature of these compounds, revealing that certain ones are edible due to their nutritious content [26]. Others have demonstrated antioxidant properties [27] or significant levels of fatty acids [28,29]. Within the Mediterranean region, six species of the *Chaetomorpha* genus are found [29]. Among the various species, *Chaetomorpha linum* (O.F. Müller) Kützing stands out as the most widely distributed and has been extensively researched from an ecological standpoint [27,30,31]. Recently, it has also gained attention for its biotechnological applications, notably in the use of its extracts for animal disease management [32,33] and in the cosmetics sector [34]. Extracts from *C. linum* have been investigated for their antibacterial, antifungal, anti-inflammatory, and antimalarial properties, in addition to showing antioxidant, antidote, antiviral, and radical scavenging capabilities [12,32,35,36]. A dietary mix of *C. linum* and *Zostera marina* has been shown to influence the growth, food consumption, and energy levels of the sea cucumber, *Apostichopus japonicus* [37]. Utilizing dietary approaches involving *C. linum* has demonstrated significant positive outcomes in aquaculture, particularly with *Labeo rohita* [38,39,40], tilapia (*Oreochromis mossambicus*) [41], catfish (*Clarias batrachus*) [42], and guppy (*Poecilia reticulata*) [43]. Additionally, *C. linum* is notably rich in chlorophyll a and b [30], which are frequently extracted from seaweeds for use as food pigments and for their health benefits [44]. The current gap between the supply and commercial potential of this seaweed presents an opportunity to market *C. linum* as an organic nutraceutical. The current pilot study seeks to assess the impact of *C. linum* algae extract supplementation on the growth, biochemical, antioxidant capacity, immunity, and immune-related gene expression of the freshwater gastropod snail, *B. bengalensis*, in the context of infection by *A. hydrophila*. Given that there has been no prior research investigating the use of *C. linum* in gastropods and other mollusks, this study offers new insights into its potential function as a natural feed supplement.

## 2. Materials and Methods

The identification of snail and algal species was carried out under expert supervision, following standardized taxonomic positions as specified in the reference checklist [45,46,47].

### 2.1. Experimental Snail

Healthy *B. bengalensis* snail adults were obtained from the local market in Dimapur, Nagaland, India. We removed sediments from the shell surface with a toothbrush and allowed them a week of acclimatization in a 250 L aerated cement tank [48,49]. Throughout the experiment, daily measurements were taken of the tank’s water temperature (26.5–28.0 °C), pH (7.6–8.0), dissolved oxygen (5.5–6.3 mg/L), ammonia (0.001–0.002 mg/L), nitrate and nitrite (<0.1 mg/L), and turbidity (0.01–0.05 mg/L), and a 12 light–12 dark photoperiod was maintained.

### 2.2. Preparation of Algae Extract

*C. linum* was collected from the Vellar estuary (Latitude 11°29′45.9″ N; Longitude 79° 46′ 25.8″ E), Tamil Nadu, India. The collected algae were thoroughly washed with seawater and tap water before being placed in a zip bag for transfer to the laboratory. Fresh algae were air-dried in the shade at room temperature for 24 h and then dried in an oven at 40 ± 1 °C. The dried seaweed was subsequently cut into small pieces (0.5–1 cm) and ground using an electric mixer, followed by sieving through a 0.5 µm filter. Algae extracts were obtained following the extraction method detailed by Widyastuti et al. [50]. The traditional method for preparing the extract involved the maceration of 250 g of dry algae in 1000 mL of 70% ethanol. The mixture was then subjected to extraction at room temperature for a duration of 72 h. Filtration of the resultant mixture was performed using Whatman no. 41 paper. Subsequently, the filtrate was transferred to a glass container and evaporated at 60 °C utilizing a rotary evaporator. The yield of *C. linum* extract was 13.92%. All experiments were performed in triplicate. The resulting residue from the extraction (CLE) was stored in a refrigerator for further use. Phytochemical screening was conducted by evaluating the coloration of phenolic compounds and assessing the chlorophyll content [50].

### 2.3. Diet Preparation

The basal diet, serving as the control diet, was formulated to maintain iso-nitrogenous (30% crude protein) levels, which agreed with the suggested nutrient values for snails [51]. The experimental diet was prepared by adding the basal diet with varying levels of *C. linum*, as detailed in Table 1. To formulate the diet, all feed components were measured, ground, and sieved through a 40-mesh screen before being carefully mixed with fish oil and a vitamin premix using a blending machine. Then, the respective algal extracts were incorporated into the basal diets (CLE0 as control; 1 g/kg for CLE1; 2 g/kg for CLE2; 3 g/kg for CLE3; and 4 g/kg for CLE4). An appropriate volume of water was then added to obtain a stiff dough. The mixture was processed into a 2 mm diameter form using a pelletizer (Junxifu, Jinan, China), followed by air-drying for 24–48 h at room temperature, and stored at −20 °C until further use.

### 2.4. Experimental Design

After a 7-day acclimatization period, 525 adult *B. bengalensis* (initial weight: 4412 ± 165.25 mg) were randomly assigned to 15 (*n* = 35 snails/tank) rectangular glass aquariums (45 L capacity), with 35 snails per aquarium. Triplicate aquariums were assigned at random to each of the five experimental diets. Each aquarium was filled with clean groundwater and contained a bottom layer of sterilized sand that was 4 cm thick. For 60 days, the snails were fed the experimental diets twice daily at 08:00 and 17:00 h, at a rate of 3% of their body weight. Any uneaten feed was collected and weighed after two hours of feeding. The amount of feed consumed by each tank was monitored daily. Every two weeks, the sand was replenished, and the tanks were thoroughly cleaned.

### 2.5. Sampling Procedure

Following 60 days in the trial, snails (*n* = 15 snails/tank) were anesthetized using an ice cube and underwent a fasting period of 24 h. The total body weight and length of each snail in each tank were measured using electronic balance and Vernier caliber. To assess the activity of digestive enzymes, three snails from each tank were randomly eviscerated, and their gut tissues were preserved at −80 °C. Additionally, hepatopancreas samples (*n* = 5 snails/tank) were rapidly frozen using liquid nitrogen and stored at −80 °C. For proximate analysis of the tissue, three snails from each tank were collected and maintained at −80 °C. According to the procedures outlined by Tachapuripunya et al. [52], snail mucus was collected at the end of the feeding trial. Ten snails from each aquarium (10 snails/tank) were bathed and cleaned with sterile distilled water before the mucus extraction process. Initially, the shell was removed with caution to avoid damaging the underlying tissue. The samples were then placed in a sterile Petri dish, after which 2 mL of distilled water was applied to the surface of the entire snail’s body. A glass stirring rod with a rounded end was employed to gently rub the snail’s skin, allowing mucus to be gradually released over a period of five minutes and subsequently dissolved in the water. The resulting mucus solutions were centrifuged at 8000× *g* for five minutes, followed by the separation of the supernatant and filtration. The mucus solution was collected in 2 mL microcentrifuge tubes and stored at −20 °C until further use.

### 2.6. Growth Performance

The following formulas were used to estimate the following measurements: viscera index (VSI), muscle tissue index (MTI), survival rate (SR), weight gain rate (WGR), and specific growth rate (SGR) [53].

WGR (%) = 100 × [(FW − IW)/IW].

SGR (% IW/day) = 100 × (ln FW − ln IW)/days.

DGR (g/day) = FW − IW/days.

SR (%) = 100 × (final number of snails/initial number of snails).

VSI (%) = 100 × (visceral weight/body weight).

MTI (%) = 100 × (soft muscle weight/body weight).

FW: final weight, IW: initial weight

### 2.7. Proximate Composition

The proximate chemical composition of snail samples, CLE, and experimental diets was examined using the procedures of the Association of Official Agricultural Chemists (Association of Official Analytical Chemists AOAC) [54]. In brief, samples were dried in an oven (Model: LHAON-95; Labindia Instruments, Mumbai, India) at 105 °C until they obtained an equivalent weight that allowed them to quantify the moisture content. The total amount of ash was determined by burning it in a muffle furnace (Model: TC303, Hasthas Scientific Instruments India (P) Ltd., Chennai, India) at 550 °C for 8 h. The standard Kjeldahl method (Model: STXKJD3; Stericox India Private Ltd., New Delhi, India) was used to assess crude protein. To determine the crude lipid, the Soxhlet extractor (Model: ACM-54097-W; ACMAS Technologies Pvt. Ltd., Sonipat, India) method was used.

### 2.8. Digestive Enzymes

The frozen gut samples were homogenized and centrifuged at 7500× *g* for five min in 0.9% precooled and sterilized normal saline (1:9 intestinal tissue to saline ratio). Enzyme activity was then measured in the supernatant after the sample was centrifuged for 10 min at 2500× *g* [55]. A mixture of 50 µL of tissue homogenate with 250 µL of a 1% soluble starch solution was incubated for 30 min at 37 °C to assess amylase activity. Before measuring absorbance at 540 nm, 0.5 mL of 1% dinitro-salicylic acid was added to the mixture, which was then heated for 5 min, cooled, and diluted with 5 mL of distilled water [56]. Following the methodology of Rúa et al. [57], lipase activity was evaluated. Specifically, 20 µL of the sample was combined with 60 µL of p-nitrophenyl butyrate (50 mM) and 1 mL of buffering solution (50 mM Tris-HCl, pH 8.0, containing 4% ethanol) to create the reaction mixture. The hydrolysis rate of p-nitrophenyl butyrate (p-NPB) was measured at 405 nm in five-minute intervals. The pepsin activity in gut extracts was determined using the method described by Anson [58], employing a substrate of 2% hemoglobin in 0.06 N HCl, and the activity was assessed based on the approach outlined by Natalia et al. [59]. The activities of amylase, lipase, and pepsin enzymes were reported as µmol min^−1^.

### 2.9. Antioxidant Status

The activities of superoxide dismutase (SOD), catalase (CAT), and malondialdehyde (MDA) in homogenate samples of the hepatopancreas were assessed using diagnostic reagent kits, following the manufacturer’s instructions (Nanjing Jiancheng Biological Engineering Institute, Nanjing, China) as outlined by Chelladurai and Maran [8]. SOD activity in the tissue extract was evaluated via the pyrogallol auto-oxidation method involving superoxide radicals, with results reported as U/mg protein [60]. The catalase activity was determined by tracking the reduction in absorbance of H_2_O_2_ at 240 nm, and the results were expressed as U/mg protein/min [61]. Lipid peroxidation was quantified by measuring the formation of MDA according to the method described by Ohkawa [62], and results were reported in nmol/mg protein of MDA formed.

### 2.10. Immune Parameters

#### 2.10.1. Hepatopancreas Immunity

For the assessment of acid phosphatase (ACP) and alkaline phosphatase (AKP) enzyme activities, hepatopancreatic tissues were weighed, pooled, and homogenized with 0.9% cold saline (1:9 ratio; *w*/*v*) to obtain a 10% homogenate [63]. The homogenate was then centrifuged at 2800 rpm for 15 min at 4 °C, and the supernatant was collected for the following assays. Biochemical assays were conducted using commercial reagent kits obtained from Nanjing Jiancheng Bioengineering Institute, Nanjing, China, in accordance with the manufacturer’s guidelines. The use of ACP as a marker enzyme for lysosomal membranes and AKP as an apical membrane enzyme was evaluated using the procedures outlined by Pennington [64] and Bretaudiere et al. [65], respectively. The optical density (OD) was measured at 400 nm for AKP and 405 nm for ACP, using a UV-visible spectrophotometer (Systronics, Ahmedabad, India). Enzyme activity was reported as U/mg protein per minute.

#### 2.10.2. Skin Mucus Immunity

A turbidimetric assay, as described by Sankaran and Gurnani [66] with minor modifications, was used to estimate mucus lysozyme activity. In brief, *Micrococcus lysodeikticus* (0.3 mg/mL) bacteria were dissolved in a potassium phosphate buffer (66 mM, pH 6.4) and incubated at 30 °C for 5 min. Then, 10 µL of snail mucus was added to the 96-microplate wells, followed by the addition of 175 µL of the bacterial suspension. The absorbance was measured at 450 nm, with readings taken at 0 and 10 min. One unit of lysozyme activity was defined as a decrease in absorbance of 0.001 min^−1^. The snail mucus protein concentration was measured using the Bradford assay [67], with bovine serum albumin (BSA) serving as the standard. Mucus samples and standard solutions, ranging from 0 to 1.41 mg/mL, were prepared in triplicate and added to 250 mL of Bradford reagent. The mixtures were incubated for five minutes at room temperature. The optical density (OD) was measured at 596 nm using a microplate reader. The results are expressed as mg protein/mL of sample.

### 2.11. Immune-Related Gene Expression Studies

Total RNAs were extracted from the hepatopancreatic tissues of all groups utilizing the RNAiso plus Kit (Takara Bio, Shiga, Japan). To eliminate genomic DNA contamination, the RNA samples were digested with DNase I (TaKaRa). Each 2 μg of RNA was reverse-transcribed into first-strand cDNA using the RT reagent kit (Takara) [68].

PCR assays were conducted using a SimpliAmp Thermal Cycler, Thermo Fisher Scientific (Model No: A24812, Waltham, MA, USA) System with a reaction volume of 20 μL. This volume comprised 10 μL of Taq II (Takara Biotechnology), 0.5 μL each of forward and reverse PCR primers (10 μM), 2.5 μL of cDNA template, 0.5 μL of ROX Dye II (Takara), and 6.5 μL of RNase-free water. Each reaction was performed in triplicate. The qPCR amplification protocol included an initial step at 95 °C for 30 s, followed by 40 cycles of 95 °C for 5 s and 60 °C for 30 s. In this study, three immune-related genes were targeted as follows: *muc-5ac* (mucin-5AC), *cyc* (cytochrome C), and *acp* (acid phosphatase-like 7 protein). The primer sequences for these genes were designed using Primer and are presented in Table 2. The β-actin gene served as an internal control for normalization. Relative expression levels were calculated using the 2−ΔΔCt method, as previously described by Livak and Schmittgen [68]. The PCR efficiency values and standard curve for each primer was mentioned in the Supplementary File (Table S1 and Figure S1). 

### 2.12. Bacterial Challenge

The origin and cultivation of *Aeromonas hydrophila* strains were in line with prior research [39,43]. For semi-lethal infection tests, live suspensions of *A. hydrophila* were injected into the intramuscular cavity of snails to establish the optimal concentration of the bacterial solution (1 × 10^7^ CFU/mL) as determined by Chelladurai and Maran [8]. Sixty days after the treatment, the remaining adult *B. bengalensis* in each tank continued to receive the respective test diets. Twelve snails were intramuscularly injected with 0.1 mL of virulent *A. hydrophila* suspension (1 × 10^7^ CFU/mL), while a control group received 0.1 mL of phosphate-buffered saline through the same injection method. A 14-day challenge test was performed, with daily monitoring of cumulative mortality.

### 2.13. Statistics

First, the normality and homogeneity of data were evaluated by Kolmogorov–Smirnov and Leven analyses, respectively. Then, data was evaluated using one-way ANOVA, followed by Duncan’s multiple range test (DMRT). A *p*-value < 0.05 was judged significant (SPSS Version 21, IBM Corp., Armonk, NY, USA). Additionally, orthogonal polynomial regression analysis was employed to evaluate the relationships between physiological responses and dietary CLE concentrations.

## 3. Results

### 3.1. Growth, and Feeding Performance

The highest and lowest growth parameters were in CLE3 and control, respectively, and the other groups showed intermediate values (Table 3, *p* < 0.05). The highest and lowest final weight (6514.33 vs. 5652.33 mg), shell length (31.03 vs. 25.33 mm), shell width (23.66 vs. 18.33 mm), and SGR (0.67 vs. 0.47% IW/day) were in the CLE3 and control groups, respectively. In addition, growth parameters showed both linear and quadratic responses to dietary CLE levels (*p* < 0.05). Somatic indices, including VSI and MTI, were not affected by dietary CLE level; however, MTI showed a positive linear response to dietary CLE concentration. Survival rate was not affected by the experimental groups.

### 3.2. Proximate Composition

Table 4 displays the proximate composition of adult snail muscle. This study found no differences in the whole-body crude protein, lipid, moisture, and ash content between snails fed the experimental diets (*p* > 0.05).

### 3.3. Digestive Enzymes

The highest and lowest pepsin activities were in CLE3 and control, respectively, and showed both linear and quadratic responses to dietary CLE level (*p* < 0.05, Table 5). Supplementing the diet with CLE increased amylase activity compared to the control group, and showed both linear and quadratic responses to dietary CLE level (*p* < 0.05). The highest and lowest lipase activities were in CLE3 and control, respectively, and showed both linear and quadratic responses to dietary CLE level.

### 3.4. Antioxidant Activity

**Figure 1:**
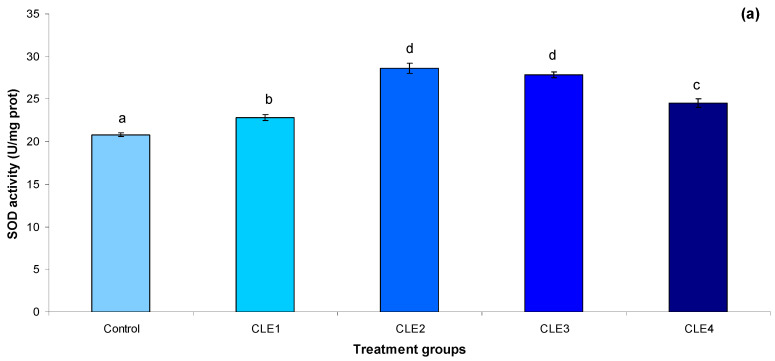

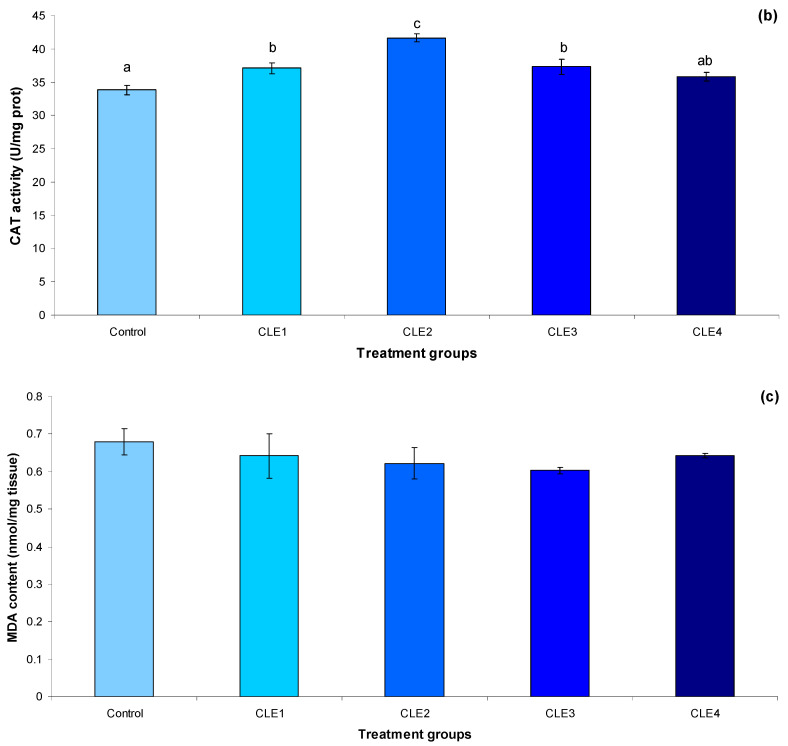
(**a**–**c**). Effects of *C. linum* extract (CLE) levels on antioxidant levels of freshwater snail, adult *B. bengalensis*, after 60 days. Data expressed as mean ± SE. Different letters above the bars show significant differences among the treatments (*p* < 0.05). (**a**): SOD (sodium dismutase) activity; (**b**): CAT (catalase) activity; (**c**): MDA (malondialdehyde) activity.

Supplementing diet with CLE increased SOD activity in the snail’s hepatopancreas compared to the control group, and showed both linear and quadratic responses (Figure 1a). The highest and lowest CAT (Figure 1b) activities were in CLE2 and control, respectively, and showed a quadratic response to dietary CLE level (Table 6). MDA level (Figure 1c) in hepatopancreas was not affected by dietary CLE level.

### 3.5. Immune Responses

**Figure 2:**
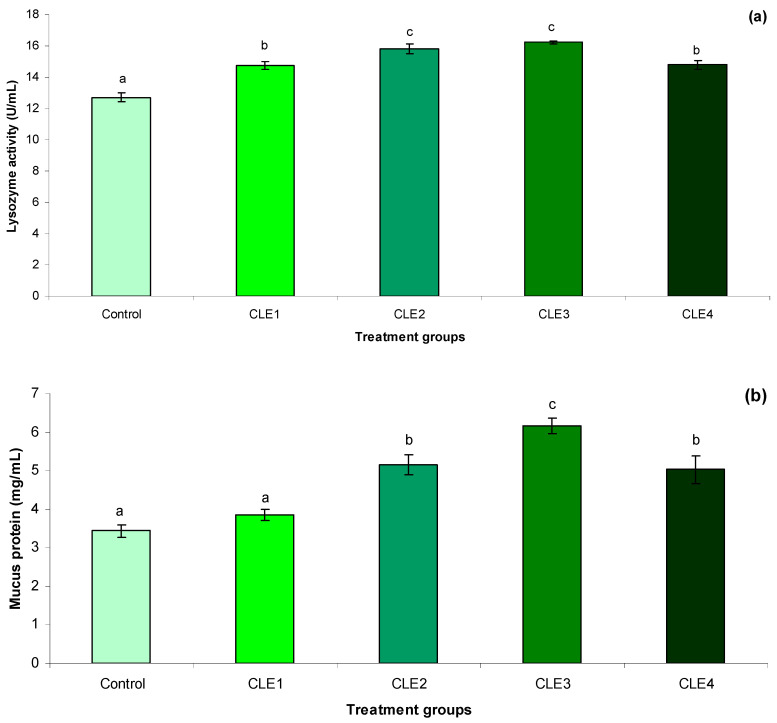

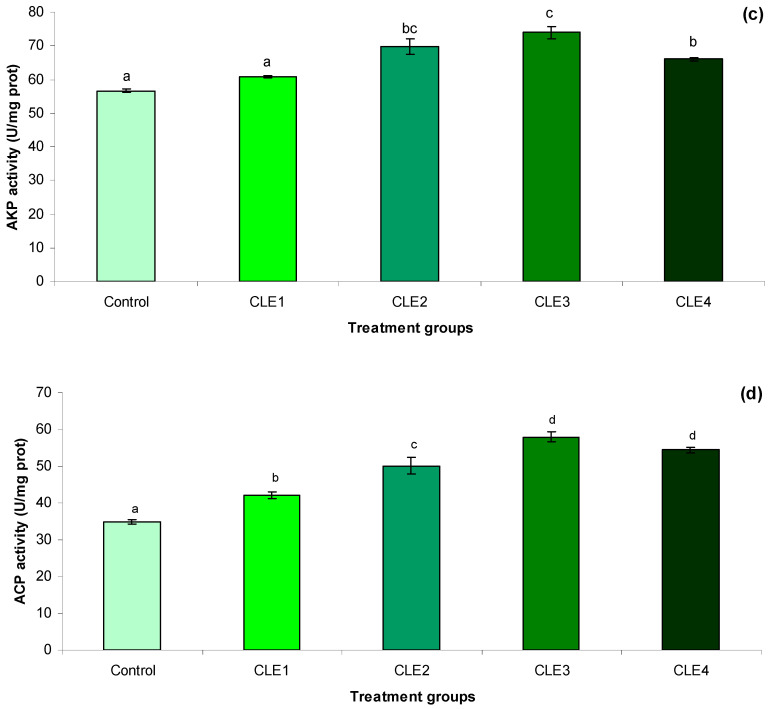
(**a**,**b**) Effects of *C. linum* extract (CLE) levels on immune responses in mucus and (**c**,**d**). Immune responses in hepatopancreas in freshwater snails, adult *B. bengalensis*, after 60 days. Data expressed as mean ± SE. Different letters above the bars show significant differences among the treatments (*p* < 0.05). (**a**): Mucus lysozyme activity; (**b**): mucus protein; (**c**): hepatopancreas alkaline phosphatase (AKP) activity; (**d**): hepatopancreas acid phosphatase (ACP) activity.

Mucus lysozyme activity (Figure 2a) increased in snails fed CLE-supplemented diets compared to the control, and showed both linear and quadratic responses to dietary CLE (Table 6). Mucus protein level (Figure 2b) and hepatopancreas AKP activity (Figure 2c) in CLE3 were higher than in the control and CLE1 groups, and the other treatments showed intermediate values. The ACP activity in the hepatopancreas (Figure 2d) increased by supplementing the diet with CLE and CLE3, and the control had the highest and lowest ACP activities. There were both linear and quadratic relationships between mucus total protein, hepatopancreas AKP, and ACP activities and dietary CLE level (Table 6).

### 3.6. Immune-Related Gene Expression Studies

**Figure 3:**
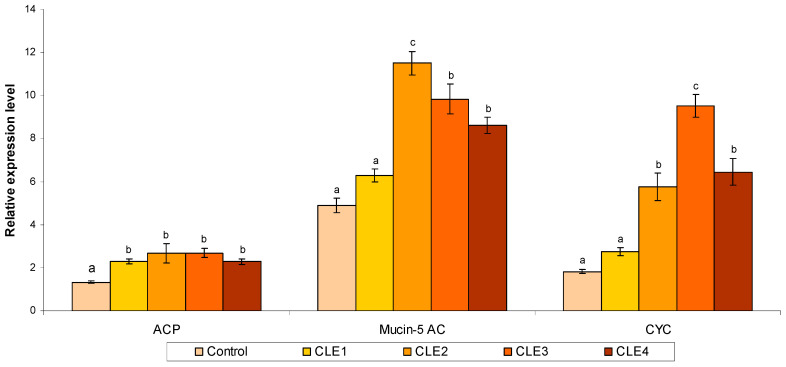
Relative mRNA (acid phosphatase-like 7 protein, mucin-5AC; CYC-cytochrome) expressed in hepatopancreas of freshwater snail, adult *B. bengalensis* fed different levels of *C. linum* extract (CLE) after 60 days. Data expressed as mean ± SE. Different letters above the bars show significant differences among the treatments (*p* < 0.05).

Figure 3 depicts the relative expression of immune gene mRNA in *B. bengalensis* hepatopancreas cells. Feeding CLE significantly increased mRNA expression of the *acp* gene in the hepatopancreas of *B. bengalensis* compared to the control (*p* < 0.05). The *mucin-5ac* and *cyc* transcription levels in CLE2, CLE3, and CLE4 were higher than those of the other groups. The immune-related genes showed both linear and quadratic responses to dietary CLE level (Table 6).

### 3.7. Challenge Test

**Figure 4:**
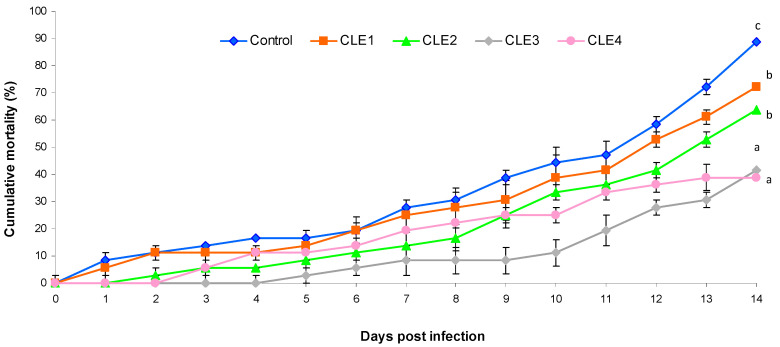
Effects of *C. linum* extract (CLE) levels on cumulative mortality of freshwater snail, adult *B. bengalensis* after 14 days post-infection. Different letters above the bars show significant differences among the treatments (*p* < 0.05).

The observed cumulative mortality was 38.8% and 41.6% when fed 3 and 4 mg/kg of CLE supplement diet for 14 days against the *A. hydrophila* pathogen. However, the control group suffered an 88.8% mortality rate (Figure 4).

## 4. Discussion

Seaweeds, referred to as marine algae, and their extracts, have gained significance in the formulation of nutraceutical products due to their high content of bioactive molecules [70]. The objective of the present study was to utilize macroalgae meals to enhance the growth of freshwater snails and to establish a connection between macroalgae and snail culture. To accomplish this, the study examined the effects of dietary inclusion of *C. linum* extract on the growth performance and feed utilization of *B. bengalensis*. In the current investigation, the edible freshwater snail, *B. bengalensis*, fed a 3 g/kg CLE-supplemented diet, showed a substantial increase in growth parameters (weight gain, SGR, and daily growth rate) compared to the control. Prior research has indicated that 150 g/kg dried *Gracilaria cliftonii* algal supplement results in improved biomass gain, specific growth rate, shell growth rate, feed conversion rate, and feed intake in greenlip abalone (*Haliotis laevigata*) [71]. The increased growth parameters in the CLE3 group were associated with the increment of digestive enzyme activity, improvement in antioxidant capacity, and immune responses in *B. bengalensis*. Previous research into the abalone, *Haliotis asinina*, indicated that receiving 2 g/kg of *G. heteroclada* supplement resulted in higher weight gain over a 45-day feeding trial [72]. A mixed seaweed diet (composed of *G. conferta* and *U. lactuca* in a 3:1 ratio) led to significantly increased shell length and weight in *H. disucss hannai* and *H. tuberculata* compared to a diet supplemented solely with *G. conferta*, suggesting that using a mixture of macroalgae provides a synergistic effect on growth [73]. In addition, a study by Palatzidis et al. [74] indicated that a diet comprising 290 g/kg *Enteromorpha linza* macroalgae enhanced both growth and feed consumption in the gastropod *Aplysia dactylomela*. Similarly, research conducted by Prepelitchi et al. [75] found that feeding *Pseudosuccinea columella* snails a diet of Spirulina (*Arthrospira platensis)* combined with lettuce leaves in a 1:1 ratio resulted in increased growth, shell length, and egg production compared to a control group. A supplementation of 3.5 g/kg of dried *Ulva expansa* demonstrated an increase in growth rate and body weight in the California horn snail, *Cerithidea californica* [76]. After a period of three months of feeding, *Babylonia spirata* exhibited significantly higher weight gain, SGR, FCR, and survival rate when supplemented with dietary concentrations of 6 and 8 g/kg *C. indica* extract [8]. The final weight, SGR, final shell length, and shell growth rate were significantly affected by the species of macroalgae meal (*Ulva* sp. and *Gracilaria* sp.) and the inclusion levels (0, 50, 100, and 200 g/kg) in greenlip abalone (*H. laevigata*) [77]. Additionally, Greenlip abalone (*H. laevigata*) fed a commercial diet exhibited improved growth and feed utilization compared to animals fed fresh macroalgae (1:1 ratio *Ulva* sp. and *Gracilaria* sp.) [78]. A comparable trend in weight gain was noted in *H. tuberculata coccinea* fed 0.2 g/kg *G. cornea* for a duration of sixty days, leading to enhanced growth [79]. These findings suggest that seaweed is a particularly effective feed additive for mollusks. However, it is noteworthy that the weight gain of snails fed a diet of 3 g/kg of CLE was higher than that observed in *B. bengalensis* in the current study. In the present research, although macroalgae extract supported substantial growth, snails fed on formulated diets demonstrated superior growth compared to those receiving macroalgae. Commercial formulated diets incorporate highly palatable and digestible ingredients, including fish meal, cereal grains, oilseeds, and pulses. These ingredients are meticulously formulated to optimize dietary energy, lipid, protein, and amino acid levels, as well as essential vitamins and minerals conducive to growth [80,81]. While the protein content of enriched macroalgae was comparable to that of commercial diets, the latter exhibited a more favorable amino acid profile relative to fresh macroalgae, which likely influenced the growth of the snails. *C. linum* is a filamentous green algae rich in polysaccharides, protein, amino acids, polyunsaturated fatty acids, pigments, phenolic compounds, and minerals [25,35]. These compounds act synergistically to stimulate metabolic and physiological processes that enhance snail growth. In our research, growth performance was relatively reduced in the CLE4 group, suggesting high inclusion levels may compromise growth performance in *B. bengalensis* as a consequence of the presence of significant amounts of antinutritional compounds in the seaweed, which can have toxic effects and hinder the absorption of essential nutrients [25,82].

Seaweed polysaccharides, including laminarin, ulvans, and fucoidan, serve as prebiotics that enhance the balance of gut microbiota [83,84]. CLE does not appear to affect *B. bengalensis’* body proximate composition. The body proximate composition of the *B. bengalensis* snail was not affected despite being fed diets with varying levels of CLE supplement. In contrast, juvenile Pacific abalone (*Haliotis discus discus*) showed a significant increase in protein content when fed diets that included 300 g/kg of *S. horneri*, compared to the control group. Furthermore, the lipid and fatty acid profiles of abalone (*H. discus discus*) can be influenced by diets that incorporate different species of seaweed and their combinations [85,86]. Research indicates that the inclusion of 100 g/kg *Gracillaria* sp. in formulated diets increases the tissue protein content in greenlip abalone (*H. laevigata*), while the incorporation of 10% *Ulva* sp. in formulated diets enhances the tissue lipid content [77]. A separate study indicated that a diet supplemented with an equal composition of *Ulva* sp. and *Gracilaria cliftonii* (1:1 ratio) resulted in enhanced levels of protein and lipids in the tissue of abalone (*H. laevigata*) compared to a control diet [78]. Seaweed polysaccharides enhance fermentation efficiency and the production of short-chain fatty acids, potentially indicating that snails utilize a greater proportion of ingested nutrients, which may result in increased biochemical accumulation in their tissues.

Intestinal digestive enzymes, primarily lipase, amylase, and protease, are crucial for the digestion and absorption of nutrients in animals. These enzymes facilitate the breakdown of externally sourced nutrients and enhance the body’s ability to absorb these nutrients. Seaweed bioactive compounds (especially polysaccharides, phenolics, and minerals) stimulate digestive enzyme secretion [87]. The snail hepatopancreas responds by increasing enzyme synthesis and secretion. We discovered that administering CLE boosted the activity of digestive enzymes in the intestine of *B. bengalensis*, which may have resulted in better growth performance in snails fed CLE-supplemented diets, particularly CLE3, which showed the highest pepsin, amylase, and lipase activities. The activation of these digestive enzymes requires cofactors, including zinc, magnesium, iron, and copper [88]. *C. linum* is also rich in these minerals [36]. When snails consume an algal diet, these trace minerals become accessible, thereby facilitating the activation of digestive enzymes and enhancing the digestive process. Feeding allicin to ivory shells (*B. aerolata*) boosted digestive enzyme activity in the hepatopancreas and intestine [55]. Thus, diets containing 3 g/kg of supplements may offer an optimal dose of CLE for enhanced digestive enzyme activity in the gut of adult *B. bengalensis*, leading to better digestion in this study. For example, protease inhibitors, which are present in various plant-based feeds, including seaweed, can hinder the activity of protease enzymes responsible for breaking down proteins into smaller peptides and amino acids. In this study, supplementing the diet with 4 g/kg CLE partially compromised digestive enzyme activity compared to that in the CLE3 group. In this context, it has been suggested that high amounts of seaweed in the diet can supply more digestive enzyme inhibitors; these inhibitors can bind to proteolytic enzymes, disrupting the usual digestive process and reducing the efficiency of digestive enzymes. This interaction may result in incomplete protein digestion, diminished nutrient absorption, and overall poorer fish performance [89]. A formulated diet containing 10% macroalgae meal (*Ulva* sp. and *Gracilaria* sp.) was found to enhance the activity of α-amylase, trypsin, and β-galactosidase enzymes in greenlip abalone (*H. laevigata*) [77]. *C. linum* is known to contain a variety of phenolic compounds, specifically phlorotannins [89,90], which have been shown to stimulate the gene expression of digestive enzymes such as amylase, protease, and lipase in the hepatopancreas and digestive gland.

Antioxidant capability was found to be directly associated with disease resistance and overall health. CAT and SOD levels are key indications of the body’s antioxidant activity. MDA is a byproduct of lipid oxidation that harms cell shape, membranes, and interior cell structures [91,92]. Algae are recognized for their ability to promote the enzymatic antioxidant pathway due to their natural antioxidant properties [93,94]. Marine algae are abundant in various antioxidants such as phytosterols, flavonoids, polyphenols, fucoxanthin, and fucoidan, which are thought to play a vital role in combating oxidation [95]. In the current investigation, antioxidant enzymes (SOD and CAT) significantly improved in snails fed CLE-supplemented diets, particularly those fed 2 g/kg CLE. This could be owing to the presence of phenolic substances such as tannins, saponins, flavonoids, or steroids. In this sense, Sun et al. [49] discovered that providing a certain amount of allicin supplement will increase SOD, CAT, and T-AOC activity while decreasing MDA levels in ivory shells. This study found that dietary CLE could increase the intestinal capacity of antioxidants in *B. bengalensis*. An earlier study observed elevated levels of antioxidants, specifically SOD and CAT, as well as total carotenoid content at 30, 60, and 90 days following supplementation with 60 g/kg *C. indica* extract, in comparison to the control group of *B. spirata* [8]. *C. linum* contains carotenoids and chlorophylls [89], which may directly neutralize reactive oxygen species in hepatopancreas tissue. This action reduces oxidative stress and helps prevent lipid peroxidation of membranes. In the present study, the administration of a 3 g/kg CLE supplement diet led to a reduction in MDA levels and an increase in SOD and CAT levels in *B. bengalensis*.

The present study shows that dietary supplementation with *C. linum* considerably improves mucosal immune responses in the freshwater snail *B. bengalensis*, as confirmed by improved levels of lysozyme, alkaline phosphatase (AKP), acid phosphatase (ACP), and mucus protein content. This research is, to the best of our knowledge, the first to document the immunomodulatory effects of an algal dietary supplement in freshwater snails, thereby presenting new standpoints on immune nutrition in gastropods. Lysozyme is a vital humoral immune enzyme in mollusks, assisting the hydrolysis of bacterial cell walls [96]. The enhancement of lysozyme activity recorded in mucus following supplementation with 3 g/kg of *C. linum* specifies an enhanced defensive ability. This enhancement may be linked to the bioactive polysaccharides and micronutrients found in *C. linum* [25,35,90,97,98], which are documented for their role in stimulating innate immune responses in aquatic invertebrates. While these effects have been widely accepted in fish and crustaceans, their statement in freshwater snails marks an important progress in the understanding of immune modulation in gastropods. Additional research indicates that a diet containing 1 g/kg of *Amphora coffaeformis* and *Scenedesmus dimorphus* resulted in enhanced hematocyte cells and differential hemocyte counts in *Biomphalaria alexandrina* snails [99]. Additionally, an extract of *Chlorella vulgaris* at a concentration of 400 mg/L was found to increase hemocyte counts in *B. alexandrina* snails [100]. Dietary supplementation with mixed macroalgae (*Ulva lactuca* and *Spyridia filamentosa*; 1:1 ratio) was found to boost respiratory burst activity, phagocytic activity, superoxide dismutase (SOD) levels, and total hemocyte count in greenlip abalone, *Haliotis laevigata* [101]. Moreover, another study specified that a 20 g/kg dietary supplement of *Schizochytrium plantensis* enhanced total hemocyte count in *H. squamata*. The considerable increase in activities of AKP and ACP further supports the immunostimulatory role of *C. linum*. The mechanisms underlying these developments are likely multifactorial. *C. linum* contains biologically active compounds, like phenolic compounds, vitamins, and crucial minerals [25], which may work as immunostimulants. Algal polysaccharides can play a role as pathogen-associated molecular patterns (PAMPs), stimulating pattern recognition receptors in molluscan immune responses, important for improved enzyme secretion and protein synthesis [102]. Also, the antioxidant compounds present in the algae may safeguard mucus-secreting cells and immune enzymes from oxidative damage, thereby supporting their metabolic activity [103]. In the current study, a 2 g/kg diet supplemented with CLE was shown to enhance mucus lysozyme activity, mucus protein levels, and AKP and ACP activity in comparison to other treatment groups.

To evaluate the effect of dietary *C. linum* supplementation on the physiological performance of freshwater snails, we further analyzed gene expression between control and experimental groups and found immune-related genes. Cellular factors, in addition to immunological components, are crucial for aquatic species’ general and particular immunity [94]. *Mucin-5ac*, a secreted mucin, is critical for mucosal immunity [104]. *Cyc* is a crucial enzyme involved in the manufacture of ATP in the mitochondria, which is released from the mitochondria due to the cell’s increased permeability upon signaling stimulation. It binds to substances like nitric oxide (NO), forming the apoptotic complex, which activates the caspase pathway and causes apoptotic cell death [105]. In this investigation, nutritional CLE supplementation dramatically increased the expression levels of the *cyc* and *mucin-5ac* genes. Sulfated polysaccharides derived from *C. linum* exhibit similarities to microbial molecules such as lipopolysaccharides (LPS) and beta-glucans [106]. Hemocytes from snails are equipped with pattern recognition receptors (PRRs) that are capable of detecting these structures. The binding of these algal polysaccharides to PRRs, including Toll-like receptors (TLRs) and *C-type lectins*, activates intracellular signaling cascades. There has been no investigation into the effects of seaweed dietary supplementation on the immune-related gene expression in snails, particularly within the context of freshwater snail research. In the present study, the supplementation of a CLE diet significantly enhanced the mRNA expression of the *acp* gene in the hepatopancreas of *B. bengalensis* when compared to the control group. The transcription levels of *mucin-5ac* and *cyc* in the CLE2, CLE3, and CLE4 groups were elevated compared to the other groups. Additionally, the immune-related genes displayed both linear and quadratic responses to varying dietary levels of CLE. Interestingly, a 200 g/kg *Chlorella* powder diet upregulated antioxidant-related genes like SOD, CAT, and GST (Glutathione-S-Transferase) in freshwater snail, *Semisulcospira coreana* [107]. In this sense, Yangthong et al. [108] found that aqueous extracts of *Sargassum* sp. at 1 and 2 g/kg of diet increased head kidney lysozyme gene expression in Asian seabass. Moreover, the expression of immune genes, including TNF-α and IL-1β, was significantly elevated in juvenile sea bream (*Acanthopagrus schlegelii*) with a 30 g/kg *S. horneri* supplement [109]. Additionally, a recent study by Liu et al. [110] indicated that administering 0.05 g/kg of dimethyl β-pripiothetine in the diet of abalone, *H. discus hannai* improved levels of *npf*, *npfr*, *nrf2*, and *ox2r genes*. In a twelve-week feeding trial, the incorporation of *Undaria pinnatifida* (β-glucooligosaccharide) at a 0.1 g/kg concentration significantly enhanced the immune system of *H. discus hannai* by upregulating immune gene expression, including *SOD*, *CAT*, *NF-Kb*, and *TNF-α* [111]. The dietary addition of *Lactobacillus plantarum* fermented algal feed (4 g/kg) led to the upregulation of immune-related genes in *H. discus hannai*, including *nrf2*, *kepa1* pathway genes [112]. In another study, a 2 g/kg of red seaweed *Chondrus crispus* sulfated polysaccharides in mussels, *Mytilus* spp. supplement resulted in increased expression of immune-related genes such as *MytM*, *DEFA1*, and *Lyz* [113].

The global aquaculture of mollusks appears to be drastically affected by bacterial pathogens and predators, resulting in substantial losses in both hatcheries and natural beds. The primary contributors to mortality outbreaks are various species of *Vibrio* and *Aeromonas*, which are recognized as significant pathogens in aquaculture [114]. Research indicates that seaweed supplements can enhance immune responses in various species of fish and shrimp. However, no published studies have directly investigated the impact of seaweed supplementation on disease resistance in freshwater snails infected with *A. hydrophila*. In the current investigation, the survival rate of *B. bengalensis* 14 days following IM injection with *A. hydrophila* revealed that snails fed diets supplemented with 3 and 4 g/kg CLE had the highest survival rates when compared to the other groups. Polysaccharides and peptides present in *C. linum* [89,90] may stimulate the synthesis of lysozyme, lectins, and anti-microbial peptides in the hepatopancreas or hemolymph. These secreted factors circulate within the hemolymph and contribute to the enhancement of the snail’s capability to neutralize bacteria and fungi. A prior investigation demonstrated that, following 7 days post intramuscular injection of *A. hydrophila*, the survival rate of the gastropod *B. spirata* was highest in specimens that were fed diets supplemented with 60 g/kg *C. indica* extract, in comparison to those on the control diet [8]. Another research indicates that a supplemental diet consisting of 1 g/kg of *Amphora coffaeformis* and *Scenedesmus dimorphus* in a 1:1 ratio increased the survival rate of *B. alexandrina* snails against *Schistosoma mansoni* infection [99]. Additionally, treatments with *C. vulgaris* extract at concentrations of 200 and 400 mg/L resulted in lower mortality rates for *B. alexandrina* snails, recorded at 130 and 170 g/kg, respectively, when compared to the control group [100]. The increase in the survival rate of spotted babylone (*B. areolate*) following exposure to *Vibrio alginolyticus* and subsequent feeding with a formulated diet is noteworthy. Specifically, *B. areolate* that received a diet enriched with 10 g/kg brewer’s yeast demonstrated increased growth alongside enhanced disease resistance [115]. The current findings suggest that a diet enriched with *C. linum* may augment the non-specific immune response in *B. bengalensis*, thereby enhancing its tolerance to *A. hydrophila* infection. This improvement in survival rate indicates that a diet supplemented with *C. linum* may represent a viable alternative to hazardous chemotherapy. According to the current research, dietary CLE improved *B. bengalensis’* non-specific immune response and triggered immune-related gene transcription that may be associated with enhancing disease tolerance capacity against *A. hydrophila* infection. This increase in survival rate suggested that a CLE-enriched diet could be a promising alternative to chemotherapy, which has many side effects.

## 5. Conclusions

The current study demonstrated that incorporating CLE at a concentration of 3 g/kg can significantly enhance growth performance, immune responses, and reduce pathogenic *A. hydrophila* infections in freshwater adult *B. bengalensis*. Additionally, measurements of digestive enzyme levels and antioxidant activity suggest that CLE can promote digestive and antioxidant capacity in *B. bengalensis*. Consequently, supplementing the diet with CLE at a level of 3 g/kg may promote overall health in this snail species. Nonetheless, further investigations into innate immunity and the proteome are required to focus on immune modulation and growth enhancement to assess the potential for commercial applications of CLE in snail farming. Furthermore, it is important to identify the active components in CLE that contribute to snail growth and immune optimization.

## Figures and Tables

**Table 1:** Composition (g /kg) of the basal and experimental diets supplemented at different levels of *C. linum* (CLE).

Ingredients	CLE0	CLE1	CLE2	CLE3	CLE4
Dehydrated cabbage powder ^a^	30	30	30	30	30
Soybean meal	25	25	25	25	25
Fish meal	10	10	10	10	10
Corn gluten meal	10	10	10	10	10
Wheat flour	15	14	13	12	11
CaCO_3_	1	1	1	1	1
Fish oil	3	3	3	3	3
Dextrin	2	2	2	2	2
Vitamin and mineral mix ^b^	4	4	4	4	4
CL extract (g/kg)	0	1	2	3	4
Chemical composition (%)					
Crude protein	30.34	30.25	30.12	30.78	30.84
Total lipid	8.43	8.53	8.41	8.73	8.64
Moisture	7.04	7.15	7.25	7.45	7.72
Ash	10.34	10.41	10.53	10.80	10.85
NFE ^c^	43.85	43.66	43.69	42.24	41.95
Phyto-chemical composition (%)	CLE				
Dry matter	15.36 ± 1.99				
Ash	20.78 ± 1.22				
Protein	15.36 ± 1.89				
Lipid	6.01 ± 0.58				
Crude fiber	12.32 ± 0.35				
Total phenolic content (mg/g)	95.34 ± 1.07				
Total chlorophyll content (mg/g)	19.38 ± 0.22				

^a^ Sigma enterprise, Pune, India. ^b^ Composition of the mixture per kg dry weight (Suplevite M obtained from Sarbahi chemicals, Wadi, Baroda-390007, India); Vit A (700,000 IU), Vit D3 (70,000 IU), Vit E (250 mg), Nicotinamide (1000 mg), Co (150 mg), Cu (1200 mg), Io (325 mg), Fe (1500 mg), Mn (1500 mg), K (100 mg), Se (10 mg), Na (5.9 mg), S (0.72%), Zn (9600 mg), Ca (25.5%), and P (12.75%). ^c^ NFE: nitrogen-free extract = 100 − (protein + lipid + moisture + ash) CLE: *Chaetomorpha linum* extract.

**Table 2:** Primer sequence for q-PCR.

Gene	Primer Sequence (5′-3′)	Product Size	Accession No.	Reference
β-actin	F: CTTGGGTATGGAATCTGCTGG R: CTTTTGCATTCTGTCAGCGAT	130	KJ871053	[69]
*MUC-5AC*	F: CAACAGGTTCCTCATCTTCG R: AAGGAGGATGCGGGAGA	110	GJDB01069827	[69]
*CYC*	F: GCAGGAAATGCCGAGAAG R AGTCTTGCGTCCAATCAGG	130	JAZBVT010000337	[69]
*ACP*	F: CAACTTCACCAAGAACACGG R: TGAGTGCTGTTGTGGATGGT	100	JALGQA010000051	[69]

Abbreviations: *MUC-5AC*: mucin-5AC; *CYC*: cytochrome C; *ACP*: acid phosphatase-like 7 protein.

**Table 3:** Growth performance of freshwater snail, adult *B. bengalensis*, fed different levels of *C. linum* (CLE).

Treatments						*p* Value		
Parameters	CLE0	CLE1	CLE2	CLE3	CLE4	ANOVA	Linear	Quadratic
IW (mg)	4412.66 ± 126.01	4439.65 ± 55.68	4472.33 ± 128.8	4360.01 ± 197.35	4319.33 ± 56.60	0.906	0.463	0.625
FW (mg)	5652.33 ± 164.29 ^a^	6107 ± 133.08 ^ab^	6350.33 ± 76.03 ^ab^	6514.33 ± 187.6 ^b^	6228 ± 61.92 ^ab^	0.010	0.011	0.001
SL (mm)	25.33 ± 0.33 ^a^	26.01 ± 0.57 ^ab^	28.02 ± 0.58 ^ab^	31.03 ± 0.57 ^b^	27.02 ± 0.51 ^ab^	0.001	0.032	0.011
SW (mm)	18.33 ± 0.33 ^a^	19.66 ± 0.31 ^ab^	21.3 ± 0.33 ^ab^	23.66 ± 0.34 ^b^	20.66 ± 0.32 ^ab^	0.001	0.007	0.001
WG (mg)	1239 ± 89.44 ^a^	1667 ± 169.45 ^ab^	1878 ± 173.16 ^ab^	2154 ± 348.25 ^b^	1908 ± 19.26 ^ab^	0.045	0.012	0.010
DGR (mg/day)	20.66 ± 1.49 ^a^	27.78 ± 2.82 ^ab^	31.3 ± 2.88 ^ab^	35.90 ± 5.80 ^b^	31.81 ± 00.32 ^ab^	0.045	0.012	0.010
SGR (%)	0.41 ± 0.020 ^a^	0.53 ± 0.050 ^ab^	0.58 ± 0.05 ^ab^	0.67 ± 0.011 ^b^	0.61 ± 0.008 ^ab^	0.010	0.015	0.021
VSI (%)	12.81 ± 0.35	12.90 ± 0.42	13.17 ± 0.15	13.36 ± 0.21	12.84 ± 0.11	0.580	0.552	0.383
MTI (%)	28.67 ± 1.41	29.50 ± 0.78	30.18 ± 0.38	30.80 ± 0.11	30.51 ± 0.15	0.336	0.039	0.088
SR (%)	94.44 ± 1.11	96.6 ± 1.92	97.7 ± 2.22	98.88 ± 1.11	97.7 ± 0.11	0.507	0.113	0.171

Abbreviations: IW: initial weight; FW: final weight; SL: shell length; SW: shell width; WG: weight gain; DGR: daily growth rate; SGR: specific growth rate; VSI: viscerosomatic index; MTI: muscle tissue index; SR: survival. Values are means ± SE of three replicates. Mean values with different letters in the same row are significantly different (*p* < 0.05).

**Table 4:** The proximate composition (wet weight basis %) of freshwater snail, adult *B. bengalensis* fed different levels of *C. linum* extract (CLE).

Treatments						*p* Value		
Parameters	CLE0	CLE1	CLE2	CLE3	CLE4	ANOVA	Linear	Quadratic
Crude lipid (%)	0.54 ± 0.003	0.55 ± 0.03	0.57 ± 0.04	0.55 ± 0.03	0.52 ± 0.004	0.637	0.660	0.272
Crude protein (%)	10.63 ± 0.52	11.46 ± 0.52	11.33 ± 0.14	11.10 ± 0.46	10.91 ± 0.07	0.612	0.879	0.344
Ash (%)	0.72 ± 0.05	0.77 ± 0.004	0.77 ± 0.006	0.76 ± 0.03	0.75 ± 0.01	0.883	0.687	0.601
Moisture (%)	83.69 ± 0.85	83.32 ± 0.69	83.53 ± 0.38	83.4 ± 0.60	84.04 ± 0.21	0.746	0.367	0.661

Values are means ± SE of three replicates.

**Table 5:** The digestive enzyme activity of freshwater snail, adult *B. bengalensis* fed different levels of *C. linum* extract (CLE).

Treatments						*p* Value		
Enzymes	CLE0	CLE1	CLE2	CLE3	CLE4	ANOVA	Linear	Quadratic
Pepsin (µmol min^−1^)	4.88 ± 0.22 ^a^	5.30 ± 0.02 ^ab^	5.95 ± 0.14 ^b^	6.54 ± 0.15 ^c^	5.84 ± 0.14 ^b^	0.001	0.002	0.001
Amylase (µmol min^−1^)	0.15 ± 0.03 ^a^	0.4 ± 0.06 ^b^	0.53 ± 0.14 ^bc^	0.62 ± 0.08 ^c^	0.50 ± 0.02 ^bc^	0.001	0.001	0.002
Lipase (µmol min^−1^)	1.07 ± 0.02 ^a^	1.2 ± 0.03 ^ab^	1.33 ± 0.10 ^ab^	1.44 ± 0.06 ^b^	1.32 ± 0.10 ^ab^	0.047	0.011	0.009

Values are means ± SE of three replicates. Mean values with different letters in the same row are significantly different (*p* < 0.05).

**Table 6:** *p* values of ANOVA and orthogonal polynomial regression in antioxidant, immune, and immune-related genes factors in freshwater snail, adult *B. bengalensis* fed different levels of *C. linum* extract (CLE).

	*p* Value		
Parameters	ANOVA	Linear	Quadratic
SOD	0.001	0.021	0.001
CAT	0.001	0.455	0.002
MDA	0.660	0.306	0.294
LYZ	0.001	0.007	0.001
TP	0.001	0.001	0.001
AKP	0.001	0.004	0.001
ACP	0.001	0.001	0.001
ACP gene	0.015	0.037	0.001
Mucin-5AC	0.001	0.012	0.001
CYC	0.001	0.001	0.001

Values are means ± SD of three replicates.

## Data Availability

Data are contained within the article.

## Supplementary Materials

The following supporting information can be downloaded at https://www.mdpi.com/article/10.3390/ani16020289/s1; Table S1: The efficiency test values for each primer pair; Figure S1: The standard curve for each primer.
