# Supplementary material for: Dietary Green-Algae Chaetomorpha linum Extract Supplementation on Growth, Digestive Enzymes, Antioxidant Defenses, Immunity, Immune-Related Gene Expression, and Resistance to Aeromonas hydrophila in Adult Freshwater Snail, Bellamya bengalensis
Source: Animals (Basel). 2026 Jan 16;16(2):289. doi: 10.3390/ani16020289 (PMC12837281; doi:10.3390/ani16020289)
Supplement: Supplementary file 1 [file animals-16-00289-s001.zip › animals-4081991-supplementary.pdf]

animals

MDPI

Article

Dietary green-algae *Chaetomorpha linum* extract supplementation on growth, digestive enzymes, antioxidant defenses, immunity, immune-related gene expression, and resistance to *Aeromonas hydrophila* in adult freshwater snail, *Bellamya bengalensis*

Hairui Yu [1,*,†], Govindharajan Sattanathan[1,2†], Mansour Torfi Mozanzadeh[3] , Pitchai Ruba Glory[4], Swaminathan Padmapriya[5], Natarajan Thillainathan[6], Ramasamy Rajesh[7], Sournamanikam Venkatalakshmi[5]**

1 Key Laboratory of Coho Salmon Culturing Facility Engineering, Institute of Modern Facility Fisheries, College of Biology and Oceanography, Weifang University, Weifang 261061, China – sattanathanphd@gmail.com (GS); Yu - yhr6003@hotmail.com
2 Department of Zoology, St. Joseph University, Chumoukedima – 797 115, Nagaland, India
3 Department of Aquaculture, South Iran Aquaculture Research Centre, Iranian Fisheries Science Research Institute (IFSRI), Agricultural Research Education and Extension Organization (AREEO), Ahwaz, Iran mansour.torfi@gmail.com
4 Department of Zoology, M. R. K. College of Arts and Science, Pazhanchanallur, Kattumannarkoil – 608 301, TamilNadu, India – slotraufrubaglory@gmail.com
5 Department of Zoology, Government College for Women (Autonomous), Kumbakonam – 612 001, TamilNadu, India. spsrijan23@gmail.com (SP) dr.s.venkatalakshmi@gcwk.ac.in (SV)
6 Department of Biotechnology, College of Science and Humanities, SRM Institute of Science and Technology, Kattankulathur 603 203, Chennai, Tamil Nadu, India. nndearar@gmail.com
7 Department of Zoology Sacred Heart Arts and Science College, Perani – 6150202, Tindivanam, Villupuram, TamilNadu, India, rajdhivo@gmail.com
* Correspondence: *Hairui Yu, yhr6003@hotmail.com ; **Govindharajan Sattanathan: sattanathanphd@gmail.com

† These authors contributed equally to this work.

Academic Editor(s): Name

Received: date
Revised: date
Accepted: date
Published: date

Copyright: © 2026 by the authors. Submitted for possible open access publication under the terms and conditions of the Creative Commons Attribution (CC BY) license.

Supplement Table S1: The efficiency test values for each primer pair

S. No	Gene	R^2 values	PCR efficiency (E)	Efficiency values (%E)
1	*β-actin*	0.996	1.89	92.3%
2	*acp*	0.996	2.02	102.6%
3	*muc-5AC*	0.990	1.94	94.1%
4	*cyc*	0.995	2.01	101.7%

https://doi.org/10.3390/ani16020289

Supplement Figure S1: The standard curve for each primer

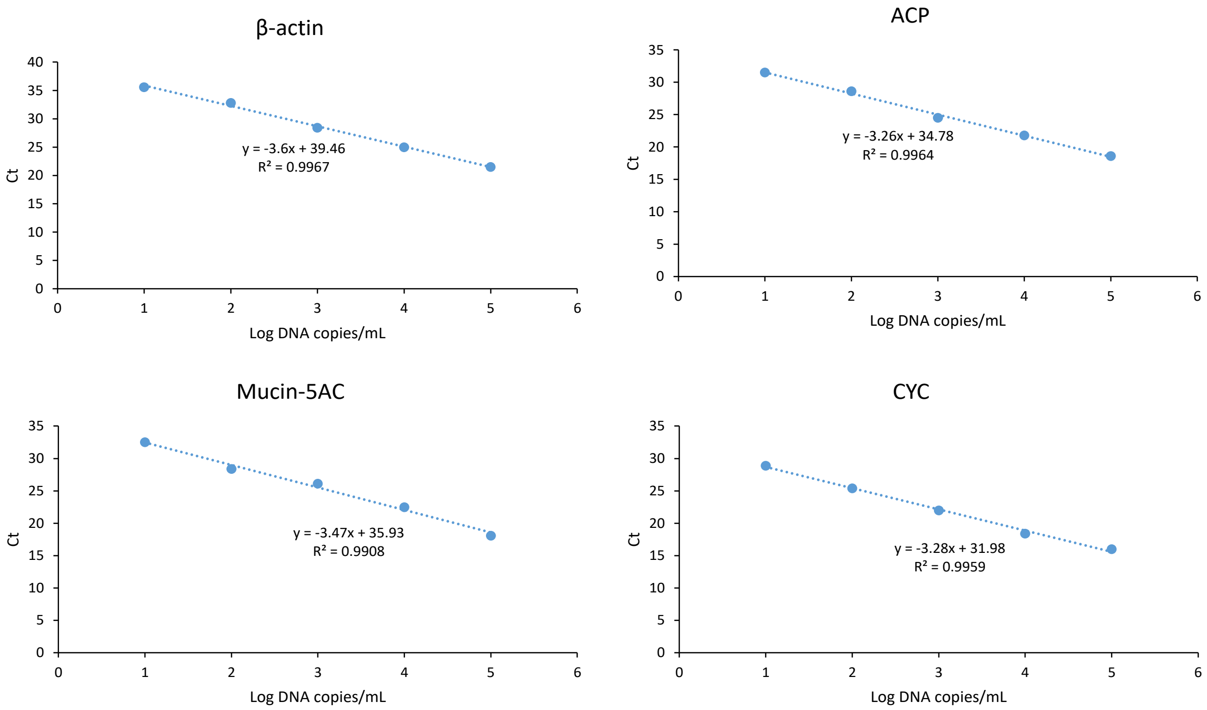

https://doi.org/10.3390/ani16020289
